# Neovascular Glaucoma Secondary to Choroidal Metastasis From Lung Adenocarcinoma: A Palliative Management Challenge

**DOI:** 10.7759/cureus.109558

**Published:** 2026-05-24

**Authors:** Nur Hanisah Mohamad Kani, Chenshen Lam, Othmaliza Othman

**Affiliations:** 1 Department of Ophthalmology, Faculty of Medicine, Universiti Kebangsaan Malaysia, Kuala Lumpur, MYS; 2 Department of Ophthalmology, Hospital Canselor Tuanku Muhriz, Kuala Lumpur, MYS

**Keywords:** choroidal metastasis, lung adenocarcinoma, neovascular glaucoma, ocular metastasis, palliative radiotherapy

## Abstract

Choroidal metastasis is the most common intraocular malignancy in adults and may present as the initial manifestation of an underlying systemic malignancy. We report a case of a 62-year-old woman who presented with unilateral progressive visual deterioration and was initially referred with a working diagnosis of ocular tuberculosis (TB). Ocular examination revealed a non-pigmented choroidal mass with exudative retinal detachment. Subsequent imaging identified a lung lesion, and a primary lung malignancy was suspected based on contrast-enhanced computed tomography of the thorax. Histopathological examination later confirmed lung adenocarcinoma. The choroidal metastasis demonstrated rapid progression, with anterior extension leading to shallowing of the anterior chamber, rubeosis iridis, and eventual development of neovascular glaucoma (NVG) with acute angle closure. The patient developed severe ocular pain with markedly elevated intraocular pressure, which was refractory to maximal medical therapy. Management was guided by the advanced stage of systemic disease and poor visual prognosis. As enucleation was declined by the patient, palliative external beam radiotherapy was performed, resulting in significant symptomatic relief. This case underscores the importance of early consideration of malignancy in atypical choroidal lesions, particularly in TB-endemic settings, and highlights the potential for rapid intraocular progression with significant complications. A multidisciplinary, patient-centred approach with emphasis on symptom palliation is essential in advanced disease.

## Introduction

Choroidal metastasis is the most common intraocular malignancy in adults, with breast carcinoma being the most frequent primary source (40-53%), followed by lung carcinoma (20-29%) [1]. Bilateral and multifocal lesions are more commonly associated with breast cancer, whereas lung cancer typically presents with a unilateral, solitary lesion. The choroid is particularly susceptible to metastatic involvement due to its rich vascular supply, which facilitates hematogenous dissemination of tumour cells [2].

The clinical presentation of choroidal metastasis is variable, ranging from asymptomatic lesions to significant visual impairment. In approximately 8-30% of cases, choroidal metastasis represents the initial manifestation of an underlying primary malignancy [3-5]. Neovascular glaucoma (NVG) is a rare but severe complication that can result in significant ocular morbidity.

We report a case of choroidal metastasis secondary to lung adenocarcinoma presenting with NVG and acute angle closure, highlighting the associated diagnostic and management challenges.

## Case presentation

A 62-year-old woman with no known systemic illness presented with a one-month history of progressive visual deterioration in the right eye, predominantly affecting the superior visual field, associated with floaters. She had no fever and denied any systemic symptoms. She was initially referred from a private center with a working diagnosis of ocular tuberculosis (TB).

On examination, the right eye visual acuity was 2/60, not improving with pinhole (N36). A grade 1 relative afferent pupillary defect (RAPD) was present, characterized by a weak initial constriction and greater redilatation of the pupil [6].

Anterior segment examination revealed a relatively white conjunctiva with a prominent sentinel conjunctival vessel (Figure 1).

**Figure 1:**
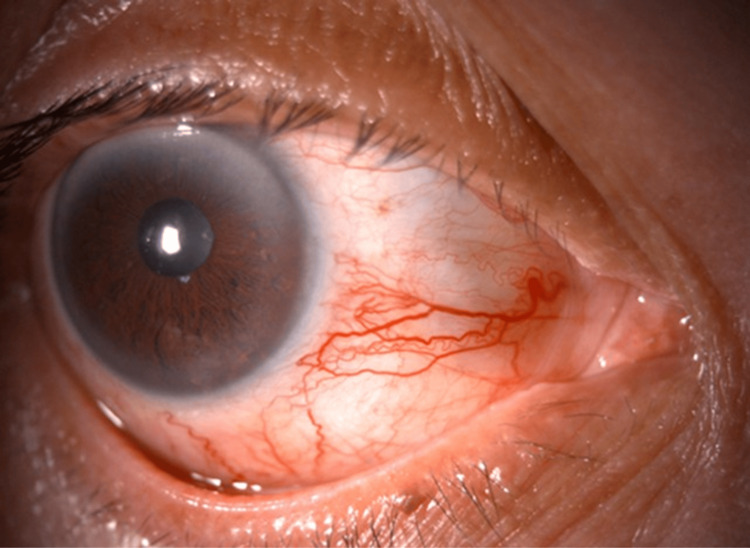
Sentinel conjunctival vessel in the right eye Anterior segment photograph demonstrating a prominent sentinel conjunctival vessel, suggestive of an underlying choroidal mass.

Fine white keratic precipitates were noted on the corneal endothelium, with occasional anterior chamber cells and 3+ cells in the anterior vitreous. The intraocular pressure (IOP) at presentation, measured using Goldmann applanation tonometry, was 16 mmHg in the right eye and 14 mmHg in the fellow eye.

Fundus examination of the right eye demonstrated a non-pigmented choroidal mass located inferonasally in the retrolental area, associated with minimal preretinal hemorrhage and vitritis. Peripheral retinal hyperpigmentation and telangiectatic vessels were also observed (Figures 2-3).

**Figure 2:**
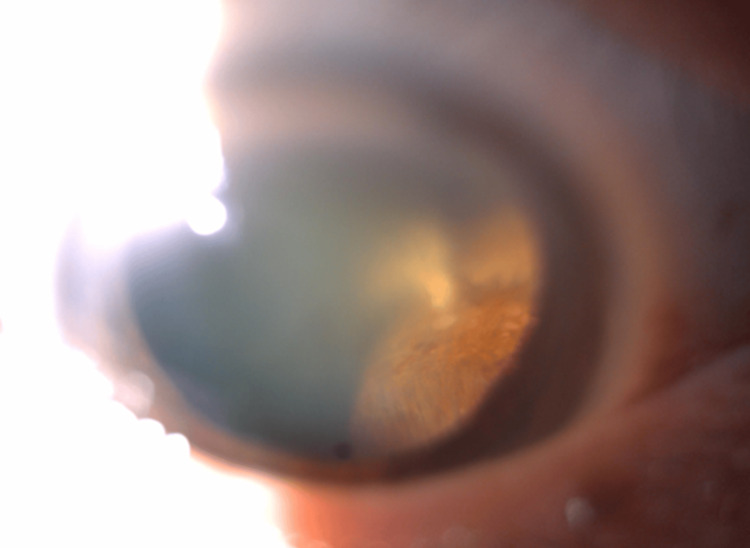
Anterior segment photograph of the right eye Choroidal mass visible through a dilated pupil

**Figure 3:**
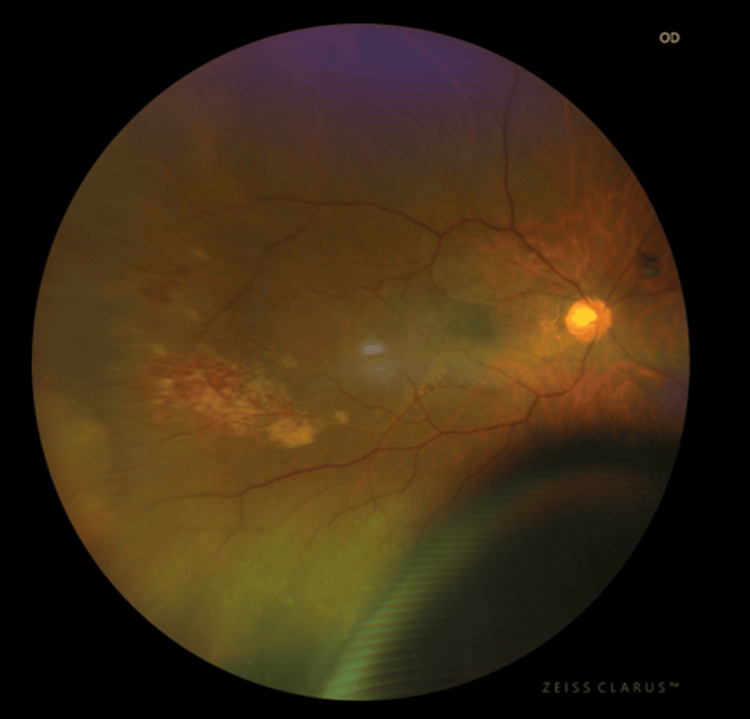
Ultra-widefield fundus image of the right eye, demonstrating a choroidal mass

The left eye examination was unremarkable. Systemically, the patient had no palpable regional lymphadenopathy or mass.

B-scan ultrasonography demonstrated a homogenous hyperechoic lesion arising from the choroid, associated with exudative retinal detachment. The lesion demonstrated low internal reflectivity without definite acoustic hollowness. Optical coherence tomography (OCT) of the macula showed the presence of subretinal fluid (Figure 4).

**Figure 4:**
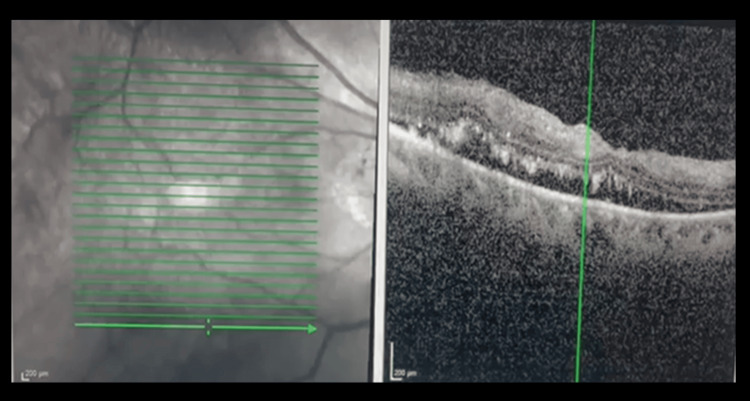
Optical coherence tomograph (OCT) of the macula of the right eye Subretinal fluid is present.

At this stage, the provisional diagnosis was amelanotic choroidal melanoma, with differentials including choroidal metastasis and ocular TB.

Further systemic evaluation revealed an incidental right lung lesion on chest radiography. Mantoux test was 9 mm with erythrocyte sedimentation rate (ESR) of 35 mm/h. All tumor markers were normal. The patient was comanaged with the Respiratory team. Contrast-enhanced computed tomography (CECT) of the thorax demonstrated a heterogeneously enhancing mass in the medial segment of the right middle lobe, measuring 3.6 × 2.4 × 2.6 cm (Figure 5).

**Figure 5:**
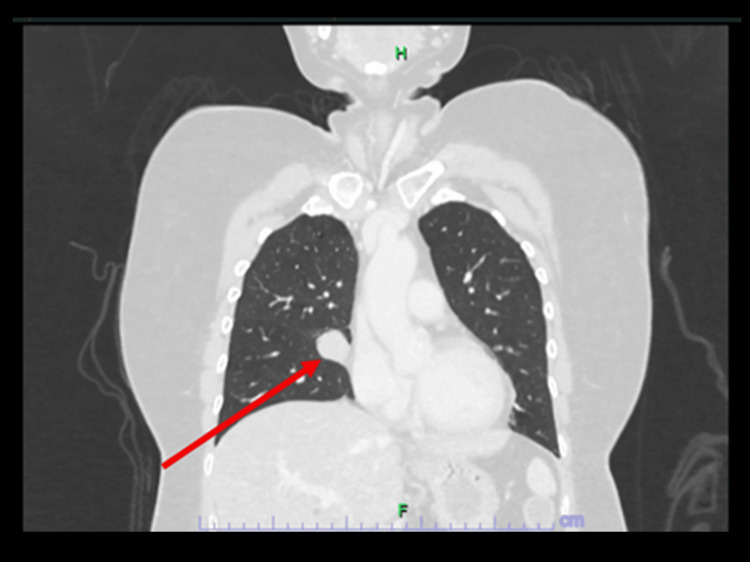
Contrast-enhanced CT of the thorax, demonstrating a right lung mass (red arrow)

Bronchoscopy showed no endobronchial lesion, necessitating CT-guided lung biopsy for histopathological confirmation. CT of the abdomen and pelvis showed no evidence of metastasis; however, positron emission tomography (PET) revealed a hypermetabolic lesion in segment III of the liver, suggestive of metastasis. Magnetic resonance imaging (MRI) of the brain and orbits demonstrated a well-defined intraocular lesion arising from the posteroinferior wall of the right globe. There was associated scleral thickening without extraocular extension. The lesion abutted the lens and displaced it superolaterally. No intracranial metastasis was identified (Figure 6).

**Figure 6:**
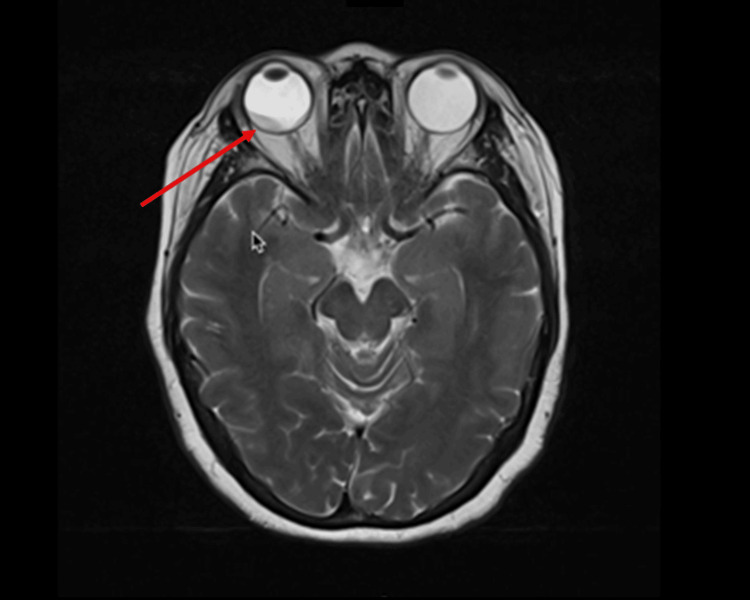
Axial MRI of the brain and orbits The finding demonstrates a right intraocular lesion (arrow), without evidence of extraocular extension or intracranial metastasis.

Despite initial stability, the lesion subsequently demonstrated rapid anterior progression, resulting in anterior displacement of the iris-lens diaphragm, generalized shallowing of the anterior chamber, and development of rubeosis iridis. The exudative retinal detachment progressed to involve the macula, with visual acuity deteriorating to hand movements. Repeated B-scan ultrasonography demonstrated an increase in tumor size (Figure 7). 

**Figure 7:**
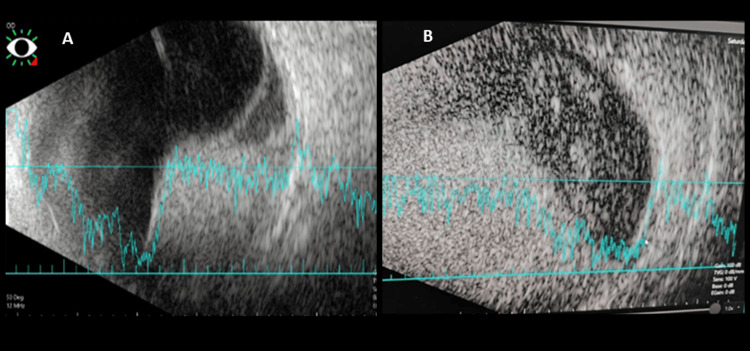
B-scan ultrasonography of the right eye (A) Initial B-scan demonstrating a choroidal mass with associated exudative retinal detachment. (B) Follow-up B-scan demonstrating interval increase in tumor size with further anterior extension.

The IOP was 14 mmHg at this stage. A prophylactic peripheral iridotomy was performed, and it was uneventful.

Over the following weeks, the patient’s condition deteriorated, and she presented to the emergency department with severe right eye throbbing pain. She was diagnosed with NVG with acute angle closure, with an IOP of 75 mmHg. Visual acuity had reduced to perception of light (PL). Examination revealed a 360° shallow anterior chamber with iridocorneal touch and extensive rubeosis iridis.

The patient was managed aggressively with topical antiglaucoma medications, oral acetazolamide, intravenous mannitol, and topical corticosteroids. Repeat B-scan ultrasonography showed an interval increase in the size of the choroidal mass with further anterior displacement of the lens-iris diaphragm. The histopathological examination (HPE) of the lung biopsy was still pending.

Due to severe refractory pain, the patient required co-management with the acute pain service (APS) team and was commenced on patient-controlled analgesia (PCA) with fentanyl, along with oral celecoxib and tramadol.

During admission, the patient developed hyphema in the setting of persistently elevated IOP. No distinct hyphema level was observed, thereby limiting visualization of the iris (Figure 8).

**Figure 8:**
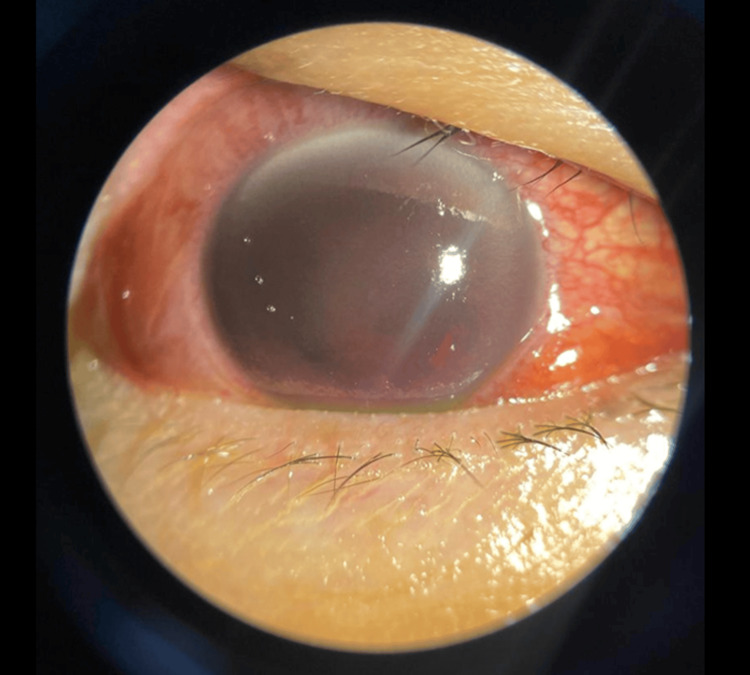
Anterior segment photograph of the right eye, demonstrating hyphema with associated corneal edema

The right eyelid was mildly swollen with conjunctiva chemosis, cornea was edematous with micro bullae. The IOP remained refractory at 50 mmHg despite maximal medical therapy.

CT-guided biopsy of the lung confirmed the diagnosis of lung adenocarcinoma. Immunohistochemistry showed tumor cells positive for CK7 and TTF-1, and negative for CK20.

Following multidisciplinary discussion involving ophthalmology, oncology, and neurosurgery teams, treatment options were carefully considered in view of the patient’s advanced disease and severe ocular symptoms.

Gamma Knife radiosurgery (GKR) was deemed unsuitable due to the anticipated delayed therapeutic response, which would not provide timely relief in the setting of acute NVG with uncontrolled pain. Enucleation was discussed as a definitive option for pain control; however, this was declined by the patient and her family.

Given the need for rapid symptom palliation, the patient was planned for palliative external beam radiotherapy to the right orbit with sparing of the contralateral eye. She received a total dose of 20 Gy in five fractions. The pain was further controlled with a regular dose of oral liquid morphine.

Following radiotherapy, the patient experienced significant improvement in ocular pain, with reduced reliance on opioid analgesia. However, visual function did not recover, and the eye subsequently progressed to a phthisical state on follow-up, at four months following completion of the palliative radiotherapy.

Molecular analysis showed no EGFR, ALK, or ROS1 mutations, with a PD-L1 tumor proportion score (TPS) of 0%. Subsequent next-generation sequencing revealed EGFR amplification. The patient was commenced on systemic therapy with gefitinib, an epidermal growth factor receptor (EGFR) tyrosine kinase inhibitor, followed by second-line chemotherapy upon disease progression.

At the latest follow-up, the right eye remained phthisical, while the patient remained clinically stable from systemic perspectives. The latest CECT of the brain, thorax, abdomen, and pelvis showed stable disease, evidenced by unchanged size and appearance of right middle lobe mass, liver, and right choroid metastases.

## Discussion

Choroidal metastasis typically occurs in the setting of advanced systemic malignancy and is associated with a poor overall prognosis [7]. Importantly, ocular manifestations may precede the diagnosis of the primary malignancy, necessitating a high index of suspicion in patients presenting with atypical intraocular lesions. Shah et al. reported that up to 44% of uveal metastases from lung cancer were identified prior to detection of the primary tumor [5]. While inflammatory conditions such as ocular TB remain important differentials in TB-endemic regions, the early inclusion of malignancy in the diagnostic consideration and prompt systemic workup were critical in enabling early recognition of an underlying malignancy in this case.

Differentiation remains challenging due to overlapping features, as both entities may present as solitary amelanotic choroidal masses with exudative retinal detachment [8,9]. Furthermore, ocular TB may occur in the absence of systemic manifestations, further complicating clinical distinction.

In our patient, the disease course was marked by rapid progression, culminating in NVG, a severe and vision-threatening complication of intraocular malignancy. Tumor-related ocular ischemia leads to upregulation of vascular endothelial growth factor (VEGF), promoting iris neovascularization and subsequent secondary angle closure [10]. The onset of NVG in this context often signifies advanced intraocular disease and represents a critical turning point, as observed in our patient, who experienced rapid clinical deterioration characterized by severe ocular pain and markedly elevated intraocular pressure.

Management at this stage requires a shift in therapeutic priorities. The management decision should be guided by the overall disease status and prognosis. Conventional antiglaucoma therapies are frequently insufficient to achieve adequate intraocular pressure control in NVG secondary to malignancy. As the disease advances, the therapeutic focus shifts from visual preservation to symptom palliation and optimization of quality of life.

In this context, the management of choroidal metastasis complicated by NVG necessitates a multidisciplinary approach, involving ophthalmology, respiratory, oncology, and palliative care teams. Treatment strategies should be individualized, balancing the limited visual potential against the need for effective pain control and systemic disease management. This case highlights the importance of early recognition of atypical presentations and the need for a holistic, patient-centered approach in managing complex intraocular malignancies.

Radiotherapy remains a key modality in the management of choroidal metastasis, particularly in patients with symptomatic ocular involvement or progressive disease. External beam radiotherapy (EBRT) is most commonly employed due to its non-invasive nature and accessibility, with high rates of tumor regression and meaningful improvement in visual and symptomatic outcomes reported in the literature [11-13]. Importantly, radiotherapy plays a predominantly palliative role, aiming to preserve visual function and alleviate pain in patients with advanced systemic malignancy. In this case, EBRT was selected for its ability to provide rapid symptomatic relief in the setting of uncontrolled NVG and severe ocular pain.

More targeted modalities, including plaque brachytherapy and stereotactic radiosurgery, such as GKR, offer improved precision and reduced radiation exposure to surrounding structures, and may be considered in selected patients with localized disease or concurrent intracranial metastases [14].

In the present case, management decisions were guided by the advanced stage of systemic disease and poor visual prognosis at presentation. With the development of NVG and rapid clinical deterioration, the therapeutic focus shifted towards symptom control rather than visual preservation. This highlights the importance of individualized treatment planning, where the potential benefits of radiotherapy must be carefully balanced against overall prognosis and patient quality of life.

What distinguishes this case is its atypical initial presentation combined with a rapidly progressive intraocular course culminating in NVG. The rapid progression to NVG underscores the aggressive nature of intraocular metastasis and the potential for significant morbidity within a short time frame. This case, therefore, emphasizes the importance of maintaining a broad differential diagnosis and the need for early reconsideration of the diagnosis when the clinical course deviates from the expected response to initial treatment.

## Conclusions

Choroidal metastasis can mimic inflammatory ocular conditions, particularly in TB-endemic regions, posing a diagnostic challenge. Early inclusion of malignancy in the differential diagnosis and timely systemic evaluation is critical. Rapid intraocular progression may result in vision-threatening complications, such as NVG, where management priorities shift from visual preservation to symptom palliation. Multidisciplinary care remains essential in optimising outcomes in advanced disease.
